# Associations of Inter- and Intraday Temperature Change With Mortality

**DOI:** 10.1093/aje/kwv205

**Published:** 2016-01-24

**Authors:** Ana M. Vicedo-Cabrera, Bertil Forsberg, Aurelio Tobias, Antonella Zanobetti, Joel Schwartz, Ben Armstrong, Antonio Gasparrini

**Keywords:** ambient temperature, diurnal temperature range, mortality, temperature variation

## Abstract

In this study we evaluated the association between temperature variation and mortality and compared it with the contribution due to mean daily temperature in 6 cities with different climates. Quasi-Poisson time series regression models were applied to estimate the associations (relative risk and 95% confidence interval) of mean daily temperature (99th and 1st percentiles, with temperature of minimum mortality as the reference category), interday temperature variation (difference between the mean temperatures of 2 neighboring days) and intraday temperature variation (diurnal temperature range (DTR)) (referred to as median variation) with mortality in 6 cities: London, United Kingdom; Madrid, Spain; Stockholm, Sweden; New York, New York; Miami, Florida; and Houston, Texas (date range, 1985–2010). All cities showed a substantial increase in mortality risk associated with mean daily temperature, with relative risks reaching 1.428 (95% confidence interval (CI): 1.329, 1.533) for heat in Madrid and 1.467 (95% CI: 1.385, 1.555) for cold in London. Inconsistent results for inter-/intraday change were obtained, except for some evidence of protective associations on hot and cold days (relative risk (RR) = 0.977 (95% CI: 0.955, 0.999) and RR = 0.981 (95% CI: 0.971, 0.991), respectively) in Madrid and on cold days in Stockholm (RR = 0.989, 95% CI: 0.980, 0.998). Our results indicate that the association between mortality and temperature variation is generally minimal compared with mean daily temperatures, although further research on intraday changes is needed.

During the last few decades, the association between daily mortality and temperature has been extensively investigated in a wide variety of settings at both the community level and the country level (1–7), and even in comparisons of countrywide estimates (8, 9). These studies have examined the associations with heat and/or cold using the mean or maximum daily temperature reached on a specific day as the exposure variable, accounting for lagged contributions. Most results have indicated a U-, J-, or V-shaped exposure-response relationship, with associations with heat lasting 3–5 days and more delayed risk of cold which extends up to 2 or 3 weeks (9, 10).

However, the extent to which the association with heat or cold is due to the high or low temperature registered on a specific day, or is in fact related to the variation in temperature between or within days, is an unresolved issue. This problem has aroused some interest lately, considering that unstable weather patterns, including sharp drops or increases in temperature, are predicted to occur more frequently in the future (11). A number of researchers have evaluated the health risk due to temperature variation using various indices, such as diurnal temperature range (DTR) (the difference between the daily maximum and minimum temperatures) as an intraday indicator and the change in mean temperature between 2 neighboring days as an interday indicator (12–17). DTR has been associated with increased risk of several health outcomes, in terms of both morbidity and mortality from different cardiovascular and respiratory causes, in recent investigations in Asia and Australia (15, 17–20). In contrast, limited investigations have been carried out on the impact of interday variation in temperature. The current evidence shows a nonlinear association, with higher risk at the extremes for both mortality and respiratory outcomes in children (12, 16, 21).

Evidence suggests that sudden changes in ambient temperature might affect population health, since the thermoregulatory system of the human body responds inefficiently to drops or increases in temperature occurring within a very narrow interval of time, with potentially different mechanisms suggested for each case (22). Individuals might feel unprepared for these sharp variations in temperature between and within days, not only physiologically but also regarding behavioral patterns (23). Increases in cardiovascular workload, increased blood pressure, severe inflammatory reactions, and infections have been suggested as potential underlying mechanisms that could worsen health status, mainly in susceptible individuals, and finally increase the probability of death (24–27).

Despite the identification of associations, clear conclusions have not been reached regarding the role of temperature variation in the overall contribution of heat and/or cold to health. This may be due in part to the lack of a conceptual model for estimating and interpreting the relative contribution of different temperature indices. In particular, the composite association with multiple temperature measures cannot be easily disentangled without some assumptions. Our objective in this paper is first to offer a more comprehensive illustration of the association of temperature with mortality, using data from 6 large cities in 4 countries worldwide with different climates, and with the definition of temperature indices based on a consistent set of assumptions. Second, we aim to compare the associations of mean daily temperature and temperature change with mortality.

## METHODS

### Collection of weather and mortality data

Daily mortality data registered in 3 European cities—London, United Kingdom; Madrid, Spain; and Stockholm, Sweden—and 3 US cities—New York, New York; Miami, Florida; and Houston, Texas—were collected for different study periods between 1986 and 2010. We evaluated total mortality in all cities, excepting for the 3 US locations, where data on deaths from nonexternal causes were available. Meteorological data on daily minimum, mean, and maximum temperatures were also obtained during the same study periods. Additional details about the characteristics of the study series are provided in Web Table 1 (available at http://aje.oxfordjournals.org/).

### Definition of temperature exposure indices

Inclusion of absolute temperature values and changes in temperature together in a regression model may produce identifiability issues, if no constraints are enforced. For instance, the same health impact on a given day can be modeled as the linear dependency of mean daily temperature on the same day and the day before (as 2 variables in a distributed lag model), or equivalently as the linear associations of mean daily temperature on 1 of the 2 days and temperature change between them. The 2 models give identical fitted values, so which model is better is not identifiable from the data. Although such complete nonidentifiability does not necessarily occur when nonlinear curves are modeled, near-equivalence of competing models can easily create problems in estimating and interpreting results from regression models that include multiple temperature indices. Below we propose definitions of temperature indicators based on more reasonable, realistic, and biologically plausible assumptions which ensure identifiability and facilitate interpretation.

The index of mean daily temperature $x_{t}^{\mathrm{mean}}$ is computed as the average between the daily maximum and the daily minimum (New York, Houston, Miami) or the 24-hour average of hourly measurements taken during day *t* (London, Madrid, Stockholm). Similarly to previous studies, the association is allowed to vary nonlinearly and with a distributed lag (8, 9).

The index of interday change is defined as the relative change in temperature between 2 neighboring days. It is assumed that the associated risk may depend on the season or the current mean temperature: For instance, a 5°C increase in temperature may be detrimental if mean daily temperature is already high, while the same increase may produce no increased risk in cold months. To implement this assumption, we built 2 independent indicators of interday temperature variation (superscript *b* indicates between-day variation in temperature): $\Delta \;x_{t}^{\;b\mathrm{inc}}$ for increase in temperature and $\Delta \;x_{t}^{\;b\mathrm{dec}}$ for decrease in temperature, computed as the difference between mean temperatures on the same day and the previous day, only if above and below the minimum mortality temperature (MMT), respectively. The city-specific MMT value was estimated from simple temperature-mortality models, as explained below in the “Statistical methods” section. The applied formulae for the estimation of interday change indicators are

(1) $$\Delta \;x_{t}^{\;b\mathrm{inc}}=\text{max}[x_{t}^{\mathrm{mean}}-\max(x_{t-1}^{\mathrm{mean}},\text{MMT}),0]$$

and

(2) $$\Delta \;x_{t}^{\;b\mathrm{dec}}=\max[\min(x_{t-1}^{\mathrm{mean}},\text{MMT})-x_{t}^{\mathrm{mean}},0],$$

where the superscript *b* indicates “between-day” and Δ indicates variation in temperature. For example, temperatures of 25°C today and 18°C yesterday, with a MMT of 20°C, correspond to $\Delta \;x_{t}^{\;b\mathrm{inc}}=5^{\circ }\text{C}$ and $\Delta \;x_{t}^{\;b\mathrm{dec}}=0^{\circ }\text{C}.$ Similarly, temperatures of 10°C and 16°C with the same MMT produce an index of $\Delta \;x_{t}^{\;b\mathrm{inc}}=0^{\circ }\text{C}$ and $\Delta \;x_{t}^{\;b\mathrm{dec}}=6^{\circ }\text{C}.$

Similarly, the change in temperature within a day, or DTR, computed as the difference between the daily maximal and minimal temperatures, might also be perceived differently depending on the current mean temperature. Therefore, we estimated 2 different intraday variation indices to better identify the association relative to the mean daily temperature: that is, DTR on hot days $\left(\Delta \;x_{t}^{w\mathrm{hot}}\right)$, considered as those days with a mean daily temperature above the MMT, and DTR on cold days, when $x_{t}^{\mathrm{mean}}$ is below the MMT $(\Delta \;x_{t}^{w\mathrm{cold}}),$ according to the formulae

(3) $$\Delta \;x_{t}^{w\mathrm{hot}}=x_{t}^{\max}-x_{t}^{\min},\;\text{if}\;x_{t}^{\mathrm{mean}}>\text{MMT},\;\text{else}\;\;0\;$$

and

(4) $$\Delta \;x_{t}^{w\mathrm{cold}}=x_{t}^{\max}-x_{t}^{\min},\;\text{if}\;x_{t}^{\mathrm{mean}}<\text{MMT},\;\text{else}\;\;0,$$

where the index *w* indicates intraday (within-day) variation (*w*hot for hot days, *w*cold for cold days), and max and min indicate the maximum and minimum temperatures on each day *t*.

### Statistical methods

Generalized linear models with Poisson regression accounting for overdispersion were applied to estimate the associations of mean daily temperature and inter- and intraday temperature variation with mortality in each city. The algebraic representation of the model is

(5) $$\log(\mu_{t})=\alpha +s(x_{t}^{\mathrm{mean}},l;\beta )+\gamma \Delta \;x_{t}^{\;b\mathrm{inc}}+\delta \Delta \;x_{t}^{\;b\mathrm{dec}}+\varphi \Delta \;x_{t}^{\;w\mathrm{hot}}+\vartheta \Delta \;x_{t}^{\;w\mathrm{cold}}+\sum_{i=1}^{p}\;f\;(z_{t}^{i};\theta ),$$

where μ*_t_* is the expected number of deaths on day *t*. The coefficients γ, δ, φ, and ϑ represent the linear dependencies of the temperature variation terms considered for the same day of exposure (lag 0). Nonlinear and delayed associations with mean temperature were estimated through distributed lag nonlinear models, where the temperature indicator $x_{t}^{a}$ was modeled through a cross-basis function (*s*) with a vector of coefficients **β** (28). Specifically, we applied a common exposure-response function for all cities consisting of a quadratic B-spline with 3 internal knots placed at the 10th, 75th, and 90th percentiles of the variable and a natural cubic spline with 3 equally spaced knots on the log scale and an intercept as the lag-response function along lags (*l*), with 21 days of lag. The other terms $f\;(z_{t}^{i};\theta )$ in the model are a natural spline function of time (8 degrees of freedom per study year) and day of the week (as an indicator variable). These modeling choices were based on previous work (8).

We first obtained the city-specific MMT using a simple model including only the cross-basis term of mean daily temperature, adopting an approach previously described (8). In the next step, all of the other indicators of temperature variation were computed and added to the model. The association with mean daily temperature was summarized as overall cumulative contributions for heat and cold at the 99th and 1st temperature percentiles, respectively, using the MMT as the reference. These were computed as the relative risks from the overall cumulative exposure-response relationships representing the net associations over the whole lag period (28). The associations with inter and intraday temperature variation was expressed as the relative risk per change in the median value.

### Sensitivity analysis

We performed several sensitivity analyses in order to check the consistency of the results obtained in the main analysis. We restricted this assessment to the London data, to obtain simpler and more easily interpretable results. The association estimates for mean daily temperature and inter- and intraday variation in temperature were again estimated, changing the number of degrees of freedom (6 df, 10 df) used to control for the time trend and the knot placement (3 internal equally spaced knots in the range of the variable) in the exposure-response function of the cross-basis term of mean temperature. In an additional sensitivity analysis, we obtained the temperature-mortality association estimates without including the temperature variation terms in the model. We also estimated the risk estimates for nonexternal mortality instead of total mortality, since 3 of the 6 cities included data on deaths from these causes only. We explored the associations with extreme interday and intraday changes by restricting the definitions of indices to days with temperature variation above their 95th percentiles. Finally, we obtained the estimates of the temperature indicators including the mean daily relative humidity in the model as a natural spline function with 3 degrees of freedom.

The R code (R Foundation for Statistical Computing, Vienna, Austria) and data used to reproduce the analysis for London are available on the personal Web page of the last author (www.ag-myresearch.com).

## RESULTS

Table 1 presents the 6 city-specific series of daily mortality and mean temperature data. The number of years included in each study period ranged from 22 (New York, Miami, Houston) to 14 (London). As expected, the median number of deaths per day was higher in the larger cities of London (161 deaths/day) and New York (169 deaths/day). A large variability in the mean temperature distribution was observed among cities, with median values ranging from 6.8°C (Stockholm) to 25.8°C (Miami). New York showed higher within-city variability, with mean daily temperatures ranging from −16.4°C to 34.4°C.

**Table 1.** Daily mortality and mean temperature data for 6 cities in a study of associations of mean daily temperature and temperature change with mortality, 1985–2010

Study Site	Study Dates	Daily Mortality^a^			Mean Daily Temperature, °C				
		No. of Deaths	Median	Range	Minimum	25th Percentile	Median	75th Percentile	Maximum
London, United Kingdom	1993–2006	845,215	161	99–353	−3.1	7.5	11.5	16	29.2
Madrid, Spain	1990–2010	577,016	74	39–256	−1.8	8.9	14.2	21.6	32.4
Stockholm, Sweden	1990–2010	201,197	26	9–51	−21.5	1.2	6.8	13.9	26.8
New York, New York	1985–2006	1,367,085	169	101–290	−16.4	5.8	13.3	21.7	34.4
Miami, Florida	1985–2006	372,130	46	23–85	3.3	23.1	25.8	28.1	31.4
Houston, Texas	1985–2006	366,340	46	18–82	−8.1	12.3	22.2	27.5	33.3

^a^ For London, Madrid, and Stockholm: total deaths (all causes); for New York, Miami, and Houston: deaths due to nonexternal causes only.

Summary statistics for variables specifying temperature changes are reported in Table 2. Miami and Houston experienced a higher percentage of days with an increase in temperature above the MMT between 2 neighboring days (between 25% and 40%), whereas in Stockholm almost half of the days included in the study period registered a decrease in temperature below the MMT. New York and Houston showed the sharpest interday increase (maximum: 7.2°C) and decrease (15.3°C) in temperature, respectively. More elevated median DTR values had been registered on hot days than on cold days in each location, except for Houston, which had the highest median DTR for cold days (12.2°C) among all cities. Madrid showed the corresponding largest median value on hot days (12.7°C). The definitions of temperature changes were based on the estimated MMT, which showed limited variation between cities, in the range 18.5°C–23°C. However, the corresponding values on a relative scale of minimum mortality percentiles were more dependent on the climate, and varied from the 25th percentile in Miami to the 94th percentile in London. Moderate-to-low correlations between the different temperature indices were observed (Table 3).

**Table 2.** Estimated Interday (Increase and Decrease) and Intraday (Diurnal Temperature Range on Hot and Cold Days) Changes in Temperature in 6 Cities, 1985–2010^a^

Temperature Measure and Study Site	No. of Days^b^	%	Change in Temperature, °C				
			Minimum	25th Percentile	Median	75th Percentile	Maximum
Interday change							
Increase in temperature, °C							
London, United Kingdom	230	4.5	0	0.4	0.9	1.7	3.6
Madrid, Spain	1,479	19.3	0.1	0.6	1.1	1.9	5.8
Stockholm, Sweden	329	4.3	0	0.4	0.8	1.4	3.7
New York, New York	1,011	12.6	0.1	0.6	1.2	2.2	7.2
Miami, Florida	3,059	38.1	0.1	0.3	0.8	1.1	5.6
Houston, Texas	1,994	24.8	0.1	0.6	0.8	1.4	5.6
Decrease in temperature, °C							
London	2,437	47.7	0	0.6	1.2	2.1	7
Madrid	2,490	32.5	0.1	0.5	1.2	2.1	8
Stockholm	3,600	46.9	0	0.6	1.4	2.5	11.1
New York	3,123	39	0.2	1.1	2.2	3.9	13.6
Miami	1,031	12.8	0.2	0.5	1.6	3.3	10.8
Houston	2,007	25	0.2	1.1	2.2	4.4	15.3
Intraday change							
DTR on hot days, °C^c^							
London	327	6.4	4.9	9.2	11.4	13.1	19.1
Madrid	2,627	34.3	3.3	11.3	12.7	13.9	19.1
Stockholm	495	6.5	0.5	8.6	10.9	13	17.6
New York	1,645	20.5	2.8	7.2	8.3	10	22.2
Miami	6,067	75.5	2.2	6.1	7.2	8.3	16.7
Houston	3,771	46.9	1.7	8.9	10.6	12.2	21.7
DTR on cold days, °C^c^							
London	4,786	93.6	1	4.8	6.6	8.6	17.1
Madrid	5,027	65.5	0.6	6	8.3	10.6	17.2
Stockholm	7,149	93.2	0.1	3.2	5.9	8.9	21.4
New York	6,383	79.4	1.1	5	7.2	9.4	23.9
Miami	1,967	24.5	2.2	7.8	9.5	11.1	18.3
Houston	4,258	53.0	1.1	8.3	12.2	15	26.7

Abbreviations: DTR, diurnal range of temperature; PMM, percentile of minimum mortality.

^a^ Temperature of minimum mortality (computed from the regression model in each city) and temperature PMM: London, 20.0°C (94th PMM); Madrid, 18.5°C (66th PMM); Stockholm, 19.0°C (94th PMM); New York, 23.0°C (80th PMM); Miami, 23.0°C (25th PMM); Houston, 23.0°C (53rd PMM).

^b^ Number of days on which the DTR differed from zero.

^c^ Days with a DTR value of zero were excluded.

**Table 3.** Correlations (Pearson Coefficients) Between Mean Daily Temperature and Inter- and Intraday Variation in Temperature (Diurnal Temperature Range) in 6 Cities, 1985–2010

	Mean Temperature	Increase in Temperature	Decrease in Temperature	DTR on Hot Days	DTR on Cold Days
London, United Kingdom					
Mean temperature	1				
Increase in temperature	0.344	1			
Decrease in temperature	−0.227	−0.111	1		
DTR on hot days	0.480	0.717	−0.165	1	
DTR on cold days	0.036	−0.347	−0.036	−0.516	1
Madrid, Spain					
Mean temperature	1				
Increase in temperature	0.486	1			
Decrease in temperature	−0.378	−0.188	1		
DTR on hot days	0.853	0.568	−0.348	1	
DTR on cold days	−0.624	−0.445	0.203	−0.824	1
Stockholm, Sweden					
Mean temperature	1				
Increase in temperature	0.299	1			
Decrease in temperature	−0.286	−0.100	1		
DTR on hot days	0.436	0.674	−0.152	1	
DTR on cold days	0.116	−0.243	−0.048	−0.370	1
New York, New York					
Mean temperature	1				
Increase in temperature	0.423	1			
Decrease in temperature	−0.357	−0.164	1		
DTR on hot days	0.648	0.626	−0.271	1	
DTR on cold days	−0.377	−0.429	0.112	−0.710	1
Miami, Florida					
Mean temperature	1				
Increase in temperature	0.300	1			
Decrease in temperature	−0.558	−0.160	1		
DTR on hot days	0.711	0.268	−0.432	1	
DTR on cold days	−0.802	−0.312	0.438	−0.843	1
Houston, Texas					
Mean temperature	1				
Increase in temperature	0.389	1			
Decrease in temperature	−0.477	−0.176	1		
DTR on hot days	0.791	0.418	−0.364	1	
DTR on cold days	−0.702	−0.396	0.272	−0.819	1

Abbreviation: DTR, diurnal temperature range.

Web Figure 1 illustrates the estimated associations between mean daily temperature and mortality, reported as bidimensional exposure-lag responses and 1-dimensional overall cumulative exposure-responses over lags 0–21 days. In general, the 6 cities displayed U- or V- shaped relationships, with increasing risks for both high and low temperatures. We observed a wide temperature interval in Miami, ranging between 23°C (25th percentile) and 28°C (75th percentile), with no association.

Summary figures for the estimated associations with mean daily temperature and inter- and intraday temperature variations are included in Table 4. Mean daily temperature was associated with a substantial increase in mortality risk, with relative risks reaching 1.428 (95% confidence interval (CI): 1.329, 1.533) for heat in Madrid and 1.467 (95% CI: 1.385, 1.555) for cold in London, reported at the 99th and 1st percentiles versus the MMT, respectively. In contrast, smaller and generally inconsistent associations were found for temperature variation indices. Results for intraday variation were difficult to interpret, with detrimental risks in London, New York, Miami, and Houston and protective associations in Madrid and Stockholm. In detail, while a relative risk of 1.010 (95% CI: 1.000, 1.020) is reported for DTR on hot days in New York, some evidence of protective associations with this index was observed on hot and cold days in Madrid (relative risk (RR) = 0.977 (95% CI: 0.955, 0.999) and RR = 0.981 (95% CI: 0.971, 0.991), respectively) and on cold days in Stockholm (RR = 0.989, 95% CI: 0.980, 0.998).

**Table 4.** Estimated Relative Risk of Mortality Associated With Mean Daily Temperature and Inter- and Intraday Variation in Temperature in 6 Cities, 1985–2010^a^

	London, United Kingdom		Madrid, Spain		Stockholm, Sweden		New York, New York		Miami, Florida		Houston, Texas	
	RR	95% CI	RR	95% CI	RR	95% CI	RR	95% CI	RR	95% CI	RR	95% CI
Mean daily temperature												
Heat	1.239	1.183, 1.297	1.428	1.329, 1.533	1.124	1.033, 1.223	1.267	1.212, 1.323	1.077	0.979, 1.185	1.026	0.955, 1.102
Cold	1.467	1.385, 1.555	1.280	1.202, 1.362	1.164	1.023, 1.323	1.189	1.127, 1.255	1.171	1.097, 1.250	1.256	1.172, 1.346
Interday change in temperature												
Increase in temperature	1.004	0.992, 1.017	1.003	0.995, 1.010	0.985	0.964, 1.008	0.997	0.992, 1.002	1.004	0.998, 1.010	0.998	0.992, 1.004
Decrease in temperature	0.999	0.995, 1.002	1.002	0.997, 1.006	1.000	0.994, 1.006	0.999	0.996, 1.002	1.001	0.993, 1.009	0.997	0.991, 1.002
Intraday change in temperature												
DTR on hot days	1.010	0.985, 1.035	0.977	0.955, 0.999	0.984	0.943, 1.026	1.010	1.000, 1.020	1.005	0.991, 1.019	1.005	0.990, 1.021
DTR on cold days	1.001	0.994, 1.008	0.981	0.971, 0.991	0.989	0.980, 0.998	1.003	0.998, 1.009	1.004	0.985, 1.023	0.998	0.986, 1.010

Abbreviations: CI, confidence interval; DTR, diurnal temperature range; RR, relative risk.

^a^ Mean daily temperature associations are summarized as overall cumulative contributions for heat and cold at the 99th and 1st temperature percentiles, respectively, with the city-specific temperature of minimum mortality as the reference category. Estimates of inter- and intraday variation in temperature are expressed as the RR per change in the median value.

We did not observe any noticeable change in the relative risk estimates from London obtained in the different sensitivity analyses (Web Table 2).

## DISCUSSION

Here we have proposed a novel modeling strategy for characterizing the relationship between temperature and mortality, assessing the contributions of different temperature indices. Our approach allows the relative associations of absolute temperature and within- and between-day changes in temperature with mortality to be disentangled and compared, thus providing a more detailed picture of the association. Our findings showed that the overall mortality risk in these cities was almost entirely determined by mean daily temperature, while temperature change played a relatively minor role.

The different and conflicting conclusions of previous studies on inter- or intra-day temperature variation may be explained by the use of ad hoc modeling strategies, which in particular did not address identifiability issues arising when modeling effects of variation indices in the presence of mean daily temperature with distributed lags (29–31). Although some of these investigations controlled for mean daily temperature, this has been modeled with limited flexibility, particularly with regard to lag structure (12, 31, 32). In contrast, the modeling approach we propose entails definitions of temperature variation indices based on a set of simple but realistic assumptions. This method provides a straightforward framework for interpretation and better allows identifiability and comparison of contributions from multiple indices. In addition, associations with indices of temperature changes are estimated in models that finely control for the nonlinear and delayed contributions of mean daily temperature through distributed lag nonlinear models.

Our results consistently indicate very little evidence for associations between interday temperature variation and mortality in the 6 cities studied. This finding contrasts with previous work suggesting that temperature variation between neighboring days is an independent risk factor responsible for a significant contribution to the impact of temperature on mortality (21, 30, 33). However, in previous studies, this index was often defined as a simple difference between mean temperatures on 2 consecutive days, without accounting for the fact that the same change is likely to have different effects depending on the absolute temperatures (21, 33). Additionally, our strategy flexibly controls for the nonlinear and delayed associations with mean daily temperature and involves more developed definitions, separating contributions due to changes above and below a referent mean daily temperature identified by the MMT. This approach extends previous attempts based on season-specific estimates (12, 30).

Conclusions regarding the relative impact of intraday temperature variation are less straightforward. In our analysis, the association was generally inconsistent and was lower than previously reported in studies not accurately controlling for mean daily temperature (20, 29, 31). Our approach, based on the definition of intraday variation with respect to the MMT, is comparable to but extends the results of previous investigations reporting associations stratified by season (13, 20, 32), similarly to Kan et al. (29). The direction of the association was not consistent with previous studies, with protective associations for DTR on either hot or cold days in Stockholm and Madrid, although the estimated mortality risks were not as substantial as those estimated for mean daily temperature. The difference may be due to our more appropriate definition of this predictor and more accurate control for the main effect of mean temperature. In a future analysis, we plan to extend this approach to specific causes of mortality, particularly causes related to more triggering hazards such as acute myocardial infarction or sudden cardiac death, in order to obtain clearer evidence on the role of this temperature index.

Some limitations must be acknowledged. In particular, our approach limits the assessment of temperature variation to the same day of exposure, as a way to simplify the analytical strategy. Identifiability problems might arise if delayed impacts in both absolute temperature and change in temperature were simultaneously accounted for. However, we cannot disregard the possibility that temperature variation might have a long-lasting and higher association during the days following the exposure, as previous studies have attempted to model (31–33). However, it should be noted that most of these investigations have found higher or similar estimates when delayed associations were not accounted for, thus suggesting that our strategy may be appropriate (13, 31). In future studies, investigators should address this issue by considering more flexible modeling strategies that properly disentangle the immediate and delayed contributions of 2 different and highly correlated temperature indicators. In addition, while we chose 6 cities to represent different baseline weather conditions, we have clearly not fully sampled the range of possibilities, and studies of other locations are warranted, potentially giving limited external validity to our results. Finally, we do not disregard the possibility that potential temporal changes in effect estimates might have occurred due to adaptation of the population to sudden changes in temperature; this issue should be further assessed in future studies.

In conclusion, this study found that the association between mortality and inter- and intraday variations in temperature was minimal in the cities studied, and that the association can largely be defined in terms of mean daily temperature only. This evidence can help in improving the predicted health impact of temperature and the design of preventive measures to limit the associated health burden.

## Supplementary Material

Web Material
