# Supplementary material for: Associations of Inter- and Intraday Temperature Change With Mortality
Source: Am J Epidemiol. 2016 Jan 24;183(4):286–93. doi: 10.1093/aje/kwv205 (PMC4753281; doi:10.1093/aje/kwv205)
Supplement: Web Material [file supp_183_4_286__index.html]

Associations of Inter- and Intraday Temperature Change With Mortality — Web Material 

# Associations of Inter- and Intraday Temperature Change With Mortality

## Web Material

Web Material

- Web Material - Pdf file
