# Supplementary material for: Associations of Inter- and Intraday Temperature Change With Mortality
Source: Am J Epidemiol. 2016 Jan 24;183(4):286–93. doi: 10.1093/aje/kwv205 (PMC4753281; doi:10.1093/aje/kwv205)
Supplement: Web Material [file supp_kwv205_kwv205supp.pdf]

Web Table 1. Characteristics of the mortality and meteorological series

	Study Period	Mortality Data		Origin of Meteorological Data
		Origin	Causes	
London	1993–2006	United Kingdom Office of National Statistics	All causes	British Atmospheric Data Centre
Madrid	1990–2010	Spanish National Institute of Statistics	All causes	Spanish National Meteorology Agency
Stockholm	1990–2010	Swedish National Board of Health and Welfare	All causes	Swedish Meteorological and Hydrological Institute
New York, Miami, and Houston	1985–2006	US National Center for Health Statistics	Nonexternal causes only	National Climatic Data Center of the US National Oceanic and Atmospheric Administration

Web Figure 1. Association between mean daily temperature and mortality (relative risk (RR)): 3-dimensional exposure-lag-response surface (left) and overall cumulative exposure-response curve (right) over a lag of 0-21 days in each city (reference temperature value: city-specific temperature of minimum mortality)

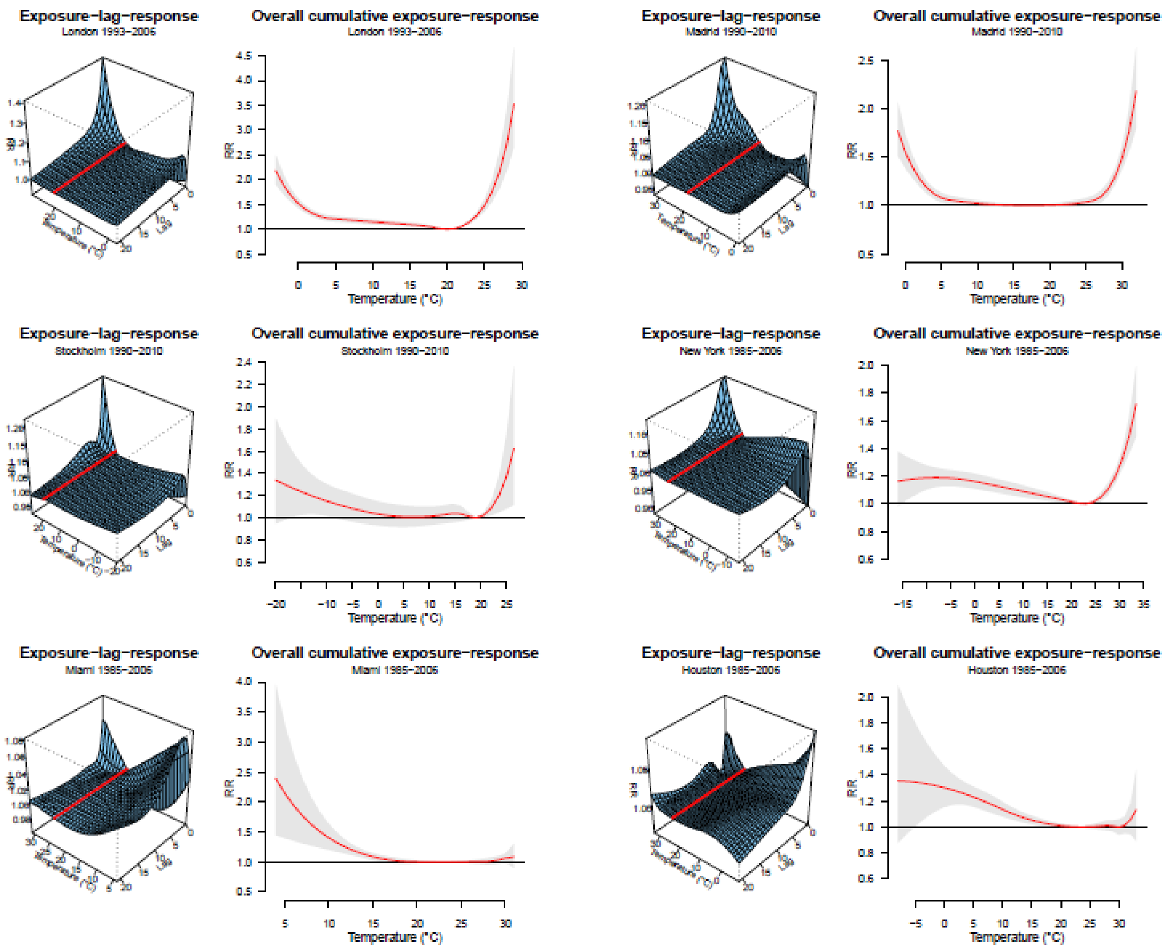

Note that the *y*-axis is rescaled to the city-specific RR range.

Web Table 2. Relative risk (and 95% confidence interval) estimates obtained from the sensitivity analyses in London

	1	2	3	4	5	6	7
Mean daily temperature							
Heat	1.221 (1.173 to 1.270)	1.235 (1.177 to 1.295)	1.245 (1.191 to 1.305)	1.230 (1.177 to 1.286)	1.238 (1.182 to 1.297)	1.250 (1.195 to 1.306)	1.234 (1.184 to 1.297)
Cold	1.559 (1.479 to 1.643)	1.394 (1.311 to 1.485)	1.453 (1.375 to 1.536)	1.441 (1.365 to 1.521)	1.475 (1.391 to 1.564)	1.462 (1.381 to 1.548)	1.461 (1.379 to 1.548)
Interday change in temperature							
Increase in temperature	1.003 (0.987 to 1.019)	1.004 (0.992 to 1.017)	1.006 (0.995 to 1.017)		1.003 (0.991 to 1.016)	1.000 (0.985 to 1.015)	1.004 (0.992 to 1.016)
Decrease in temperature	0.999 (0.995 to 1.002)	0.998 (0.995 to 1.002)	0.999 (0.995 to 1.002)		0.999 (0.995 to 1.002)	1.001 (0.997 to 1.005)	0.999 (0.996 to 1.002)
Intraday change in temperature							
DTR in hot days	1.015 (0.988 to 1.042)	1.009 (0.985 to 1.034)	0.995 (0.975 to 1.015)		1.010 (0.985 to 1.036)	1.030 (0.998 to 1.063)	1.020 (0.994 to 1.047)
DTR in cold days	1.002 (0.995 to 1.009)	1.001 (0.994 to 1.008)	1.001 (0.995 to 1.008)		1.000 (0.993 to 1.007)	1.002 (0.996 to 1.008)	1.007 (0.999 to 1.015)
MMT (MMP)	20.5 (95)	20 (94)	19.5 (92)	20 (94)	20 (94)	20 (94)	20 (94)

Abbreviations: df, degrees of freedom; DTR, diurnal temperature range; MMP, percentile of minimum mortality; MMT, temperature percentile of minimum mortality.

1: 6 df for time trend.
2: 10 df for time trend.
3: Placement of internal knots cross-basis at equally spaced in range mean temperature.
4: Model without inter- and intraday variation terms.
5: Nonexternal causes.
6: Extreme interday and intraday changes in temperature (above 95th percentile).
7: With mean daily relative humidity (natural spline with 3 df).
